# Richmond Academy of Medicine and Surgery

**Published:** 1890-08

**Authors:** W. W. Parker

**Affiliations:** President, in the Chair


### RICHMOND ACADEMY OF MEDICINE AND SURGERY.

Stated Meetings June 10 and 24, 1890, W. W. Parker, M. D., President, in the Chair.

### A PECULIAR CASE OF INDIGESTION.

Dr. J. N. Upshur reported a peculiar case of indigestion in a lady of fifty-four years, very much "run-down" from mental and physical overwork.

The peculiar feature was a severe pain, spasmodic in character, occuring periodically about every ten days. Its seat was about the pylorus and downwards and to right along the edge of the ribs. When the doctor first saw her she had three of these attacks at intervals of about twelve hours. The first he relieved in a few hours with morphia and atropia hypodermically; the two last with 1-50 gr. doses of nitro-glycerine, administering twice for second, only once for third attack. No eructation of gas and water followed the last of the three as had been the case always before. The general treatment given was a light nutritious diet, attention to bowels and a tonic of phospate of iron, quinine and strychnine. She had no recurrence of the pain. Nitro-glycerine had been suggested to the doctor's mind by the fact that the pain in its acuteness resembled the spasm of angina pectoris. He had much confidence in nitro-glycerine for the relief of the œdema, dyspnoea and cardiac distress of Bright's disease. Had tried it with much success for the temporary relief of aggravated sciatica. Though powerful in action its effects were more permanent than nitrite of amyl.

### A CASE OF LA GRIPPE.

Dr. W. W. Parker reported the case of a robust young man, afflicted with influenza, a short time ago; this being accompanied by an inflammation and considerable swelling of the muscles of the neck, and this, in turn, followed by a frightful eruption of vesicular character over the whole body, very much like chicken pox. It was particularly marked upon the lips and inner side of thigh, where it resembled, and might have been mistaken for confluent small-pox. Continued ten days or two weeks, leaving extremities first, and gradually; no fever; very slight constitutional disturbance of any kind; no itching of consequence.

SINGULAR EXPERIENCE WITH SCARLET FEVER AND MEASLES.

Dr. W. B. Grey reported, in reference to two children affected with scarlatina, aged respectively two and four years, that just about the commencement of desquamation the older one developed the eruption of measles; in four or five days the younger did the same.

Furthermore, said the doctor, about this time the father, an old man, took scarlet fever.

HÆMATOMA AURIS.

Dr. Charles M. Shields reported a case of hæmatoma auris occurring in a lawyer of about sixty years of age, and perfectly sound in mind (the trouble very rarely appearing, except in the insane.)

About a month before the appearance of the growth, the man had suddenly lost consciousness, one day, and in falling, had bruised the side of his face corresponding to the trouble. The doctor enlarged an opening; found upon anterior wall of canal about 1-2 inch from external orifice.

The cavity into which it led would hold about five or six drachms. The discharge was very offensive.

Prescribed a wash of peroxide of hydrogen. From one Saturday night until the following evening, the patient had five or six hemorrhages, losing in all, about twenty or thirty ounces of blood.

The only resource for perfect control of the flow was packing the cavity with cotton, saturated in Moswel's solution.

The doctor thought the man would recover, but with a considerable scar.

Dr. M. D. Hoge reported the case of a man, who since an attack of LaGrippe, had fallen into a state of melancholia almost amounting to insanity. He suffered excessively from nervousness and an intense pain in the head—the latter being treated successively with morphia, cocaine and bromide potassium.

He still complained of great pain in his head until one night he pounded himself over the head with a poker until he had peeled off a large piece of scalp and produced enormous hemorrhage. He then felt better. Some time after, the doctor found a sequestrum of bone (a portion of the external table) in the wound, which he removed and the part began to heal beautifully. The man was very much depressed all along; believed himself going crazy. Complained of hearing voices. The doctor reasoned him out of that state and pronounced him now upon the road to recovery.

Dr. W. W. Parker thought the hearing of voices a pretty sure sign of insanity.

S. W. HENSON, M. D.,

Reporter.

